# Childhood brain tumors: It is the child’s brain that really matters

**DOI:** 10.3389/fonc.2022.982914

**Published:** 2022-10-04

**Authors:** David A. Walker

**Affiliations:** Child Health, School of Medicine, University of Nottingham, Nottingham, United Kingdom

**Keywords:** childhood brain tumours, research outcome measures, disability, health economics, neurotoxicity, child health outcomes, disability life years

## Context of research in childhood brain tumors

The context for research into brain tumors of childhood over the past three decades has focused upon developing an understanding of the biological mechanisms of tumor formation (1). This has been pursued in the belief that it will be the key that will unlock the tumors’ vulnerability to therapeutic approaches. The “driver for change” has been improving overall survival. In childhood this has gratifyingly been associated, in high income countries (HIC), with a rise in survival rates from 40-70% (2–4). Within this statistic there are significant variations between European countries. Clinical trials have shown remarkable advances, such as intra-cranial germ cell tumors (5) and medulloblastoma (6), which have improved with combined standard approaches of well delivered chemotherapy, radiotherapy and rational approaches to surgery. Radiotherapy research and trials in the past decades have focussed upon optimising radiation doses to the tumour and surrounding brain to minimise the cognitive consequences (7).

Bio-characterization of these tumors offers hope of further stratification of outcomes with biologically targeted therapies (8). There have been surprises, such as chemo-sensitivity of low grade glioma, offering control of this early onset, self-limiting tissue growth disorder of astrocytes (9). The targeted effect of mTOR inhibitors have controlled progression of sub-ependymal giant cell astrocytoma (SEGA) complicating tuberous sclerosis (10). Bio-characterization of these benign tumors has identified single pathway mutations suitable for drug targeting (11). There have been disappointments with limited or no progress in drugs contributing to cure of ependymoma (12), diffuse intrinsic pontine glioma (DIPG) (13), atypical teratoid rhabdoid tumor (ATRT) (14, 15) and high grade glioma (HGG) (16, 17). Each of these tumor types have been bio-characterised with the intention of identifying targetable mutations to contribute to improved responses; a strategy yet to provide improvements in cure rates. These are tumors with high levels of primary drug and radiation resistance. The complexity of their diverse bio-characterization profiles, which commonly change after successive treatments, are compounded by superimposed anatomically-determined diversity of mutational patterns. This seems to undermine the rationale for bio-target driven therapies. Contemporary bioscience thinking has responded in particular to the challenges of this primary resistant group by highlighting seven research strategies to look for new therapies (1) which include:

1. Redesigning the research pipeline
2. Leveraging neuroscience research
3. Enhancing understanding of the tumor microenvironment including the blood brain barrier
4. Developing predictive models for research
5. Developing drugs for complex targets in a shifting tissue landscape
6. Developing precision medicine
7. Reducing treatments for sensitive tumor types

This comprehensive proposal is staggering in its scope and has no identifiable timetable or funding stream. The children and their families, the funders and their governments are given no guarantees on delivery or success. Is this outline a safe basis for planning a successful assault on children’s brain tumors or is it simply a backdrop for neuro-oncology research practitioners to justify anything they might suggest, in the hope that something will emerge by chance alone?

## Biology and therapy of benign versus malignant brain tumors

Biological research has clearly demonstrated that brain tumors in childhood are products of embryologically-sensitive mutations linked to age and precise neuro-anatomical locations (18–21). It is notable that over the past 4 decades there have only been 5 drugs licensed for brain tumors in adults and children, of which 4 are still in production: CCNU (22), temozolomide (23), carmustine wafers (Gliadel) (24)and everolimus (10). The first 3 are licensed for HGG, each has been selected or developed with their capacity to penetrate or bypass the blood brain barrier. Of these, only temozolomide has been licensed for children. Everolimus was licensed for SEGAs that present in Tuberous Sclerosis during late childhood and early adulthood (9). There are trials in progress to evaluate MAP Kinase inhibtors (MEKi) in low grade glioma and NF1-associated neurofibroma (25–29). It is possible therefore that MEKi will join the list of licensed drugs for children for this tumor sub-group. There are trials studying WNT medulloblastoma subtype that may offer enhanced drug penetration across the blood brain barrier and therefore greater sensitivity to standard chemotherapy (30).

What is emerging from this experience is that benign brain tumors are brain development disorders which respond to systemically administered drugs, whilst malignant brain tumors require strategies to penetrate or bypass the blood brain barrier for existing drugs to be effective. If bio-targeted drugs are to be used, a wide variety of targeted drugs will need to be tested in combinations to cover diversity of mutations and their evolution over time. Furthermore, they will need to be specifically delivered across the blood brain barrier if they are to be effective. A whole range of drug delivery techniques are emerging for further study, including intra-CSF delivery, intra-cavity/interstitial delivery, ultrasound BBB disruption, electric field therapy, immunotherapy and transmucosal delivery (31–33). They will require careful selection for study in childhood brain tumors as the biology of children’s tumor types and the state of the brain’s environment differ markedly from the adult experience and so progress will be determined by specialist paediatric centers adopting techniques for study, ideally as part of an international collaborative strategy (34).

## Selecting outcome measures as “drivers for change”

**Figure 1:**
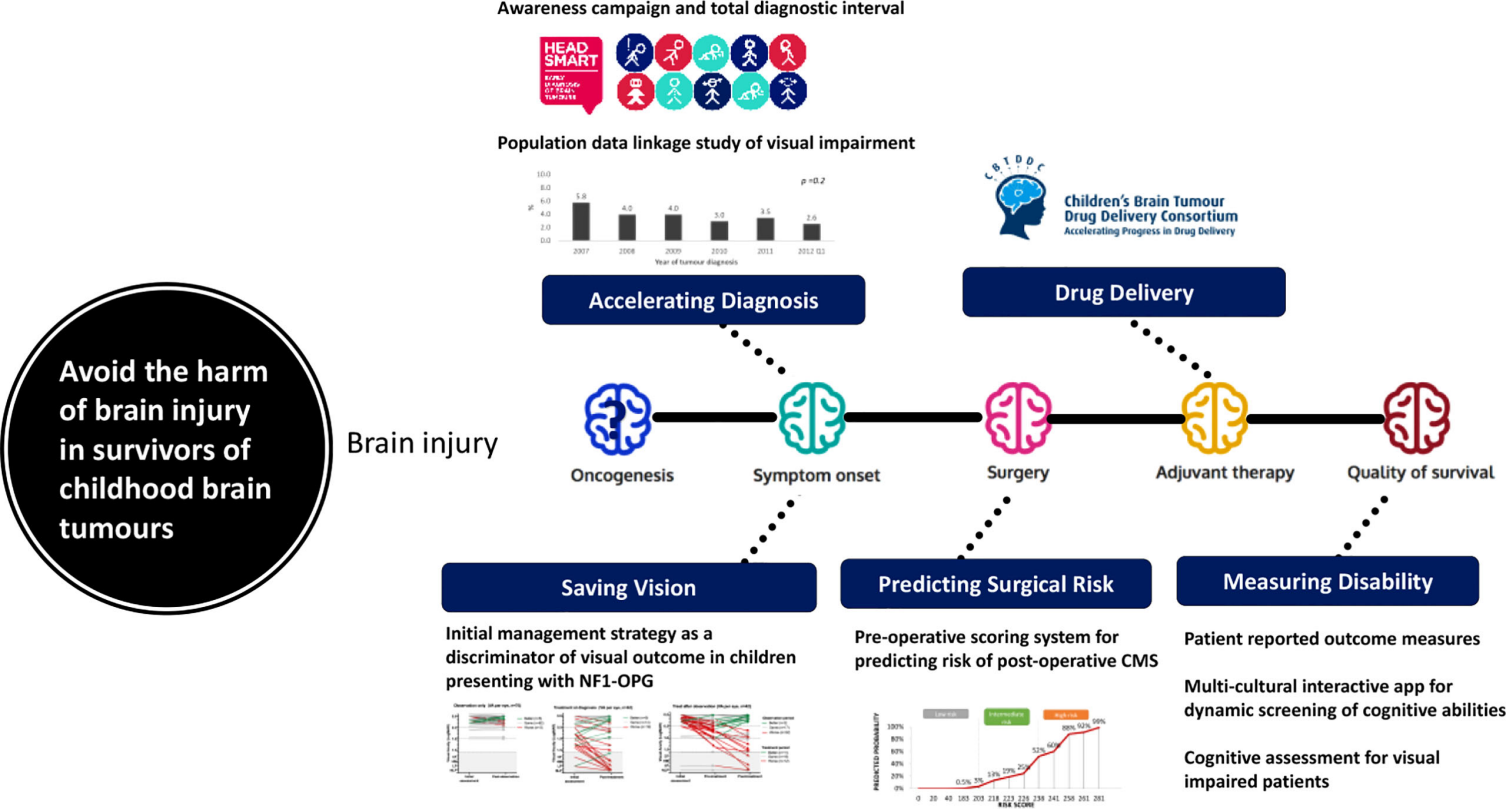
A model identifying sources of cumulative brain injury with examples of data and interventions during the management of childhood brain tumours.

The historical reliance on overall survival as the “driver for change” in the strategy has failed to recognize the incremental acquisition of brain injury by all children with brain tumor for as long as they live and therefore its major health impact for all children from diagnosis (**Figure 1**). A strategy that omits the consequences of brain injury is therefore deficient and needs review (35). The authors of the seven challenges have not identified brain injury as a target for their research priorities. Brain injury starts with symptom onset prior to diagnosis, is a recognized consequence of brain surgery, radiotherapy and drug therapy and can be exacerbated in its impact in the absence of effective rehabilitative support during childhood and adolescence (36–38). Brain injury is the experience that colors the children’s lives for as long as they live and is therefore the most important clinical target for research intervention as it applies to all children not just those who are curable.

## Strategies to minimize acquired brain injury

Accelerating diagnosis (36, 37), predicting surgical risks (39, 40) and preventing them, modifying radiation doses and techniques (41), designing trials and outcomes measures to measure neurological and disability outcomes (42) targeting drug therapy precisely (31) and promoting rehabilitative effectiveness (35) can all be considered as legitimate interventions to reduce the risk and degree of acquired brain injury, as well as other toxicities (**Figure 1**). They can be advanced as strategies immediately as they are about using real clinical data to drive change. If these strategies are to be tested, whilst they are being introduced and studied for their impact across health systems. There are promising developments in the design of trials in optic pathway glioma (26, 27) and evaluating surgical strategies in medulloblastoma (43) (M. Mynarek, personal communication). A key “driver for change” will be the selection of primary outcome measures for neurological and quality of life outcomes during childhood, adolescence and early adulthood that reflect this cumulative brain injury (42, 44).

## A global health challenge

The World Health Organisation (WHO) Child Cancer Initiative has recognized brain tumor specifically as a global priority (45). The Lancet Commission identifies the economic potential of tripling returns of investment in childhood cancer, particular in low and middle income countries (46). The material cost of acquired brain injury has been quantified by legal processes to range from £2m-26m per child. In the absence of a legal award this is the type of cost needed to support a child after treatment for brain tumor from family, health, social and community services budgets (37). The time is right therefore, to build upon the previously identified research challenges by focusing upon strategies to measure and minimize acquired brain injury in parallel with these initiatives as a sincere effort to minimize the suffering in the immediate future for the children with brain tumor and their families.

Whether the seven challenges will ever be overcome to deliver the new targeted therapies hoped for by the bio-science community remains to be seen. The children need us to deliver change soon to help them and their families have more hope for the future in the next decade. Preventing the acquisition of cumulative brain injury seems a good target for now.

## Author contributions

The author confirms being the sole contributor of this work and has approved it for publication.

## Conflict of interest

The author declares that the research was conducted in the absence of any commercial or financial relationships that could be construed as a potential conflict of interest.

## Publisher’s note

All claims expressed in this article are solely those of the authors and do not necessarily represent those of their affiliated organizations, or those of the publisher, the editors and the reviewers. Any product that may be evaluated in this article, or claim that may be made by its manufacturer, is not guaranteed or endorsed by the publisher.
